# Diet quality and carotid atherosclerosis in intermediate cardiovascular risk individuals

**DOI:** 10.1186/s12937-017-0266-1

**Published:** 2017-07-03

**Authors:** Jose I. Recio-Rodriguez, Irene A. Garcia-Yu, Rosario Alonso-Dominguez, José A. Maderuelo-Fernandez, Maria C. Patino-Alonso, Cristina Agudo-Conde, Natalia Sanchez-Aguadero, Rafel Ramos, Ruth Marti, Emiliano Rodriguez-Sanchez, Manuel A. Gómez-Marcos, Luis Garcia-Ortiz

**Affiliations:** 1 Primary Care Research Unit, The Alamedilla Health Center, Castilla and León Health Service (SACYL), Biomedical Research Institute of Salamanca (IBSAL), Spanish Network for Preventive Activities and Health Promotion (redIAPP), Department of Nursing and Physiotherapy (University of Salamanca), Salamanca, Spain; 2 Department of Preventive Medicine, Complejo Asistencial Universitario de Salamanca, Salamanca, Spain; 3 Primary Care Research Unit, The Alamedilla Health Center, Castilla and León Health Service (SACYL), Biomedical Research Institute of Salamanca (IBSAL), Spanish Network for Preventive Activities and Health Promotion (redIAPP), Salamanca, Spain; 4 Department of Statistics, University of Salamanca, Biomedical Research Institute of Salamanca (IBSAL), Spanish Network for Preventive Activities and Health Promotion (redIAPP), Salamanca, Spain; 5 Research Unit Family Medicine, Jordi Gol Institute for Primary Care Research (IDIAP Jordi Gol), Translab Research Group, Medical Sciences Department, School of Medicine, University of Girona, Biomedical Research Institute (IDIBGI), Dr. Trueta University Hospital, Catalonia, Spain; 6 Primary Care Research Unit, The Alamedilla Health Center, Castilla and León Health Service (SACYL), Biomedical Research Institute of Salamanca (IBSAL), Department of Medicine, University of Salamanca, Spanish Network for Preventive Activities and Health Promotion (redIAPP), Salamanca, Spain; 7 Primary Care Research Unit, The Alamedilla Health Center, Castilla and León Health Service (SACYL), Biomedical Research Institute of Salamanca (IBSAL), Department of Biomedical and Diagnostic Sciences, University of Salamanca, Spanish Network for Preventive Activities and Health Promotion (redIAPP), Salamanca, Spain; 8 Primary Care Research Unit. Alamedilla Health Center, 37003 Salamanca, Spain

**Keywords:** Carotid artery diseases, Food habits, Diet, Mediterranean

## Abstract

**Background:**

Diet quality indices address the diet’s complexity and are calculated by a combination of foods and/or nutrients which together represent a dietary pattern. The current study analysed the relationship between the common carotid artery intima media thickness (cIMT), the presence of plaque and the carotid target organ damage (cTOD) with the diet quality assessed through the Diet Quality Index (DQI) questionnaire in a Spanish adult population.

**Methods:**

A cross-sectional study. The target population comprised of 500 individuals aged between 35 to 74 years who had intermediate cardiovascular risk. The diet was evaluated by DQI which included beneficial and detrimental foods scored 3, 2 or 1. The total possible score ranges from 18 (the lowest quality) to 54 (the highest quality). Carotid ultrasound was used to assess the cIMT, the presence of plaque and the cTOD.

**Results:**

Among the 500 participants (mean age 60.3 ± 8.4 years), 54.4% were male. DQI mean was 40.08 ± 2.79, with no differences between men and women. The cIMT was lower in women (*p* = 0.002) and 16.6% of the participants presented plaque. No significant association was found between DQI and cIMT after adjusting by age and sex, and other confounders (*p* = 0.690). The logistic regression analysis showed no association of DQI with thickened cIMT (*p* = 0.890), the presence of plaques (*p* = 0.799) or cTOD (*p* = 0.942).

**Conclusions:**

The diet quality index was not associated with subclinical atherosclerosis in this Spanish population at intermediate risk of cardiovascular disease.

**Trial registration:**

ClinicalTrials.gov; Identifier: NCT01428934.

## Background

The measurement of common carotid artery intima-media thickness (cIMT) allows the detection of thickening of the artery wall during the initial phases of atherosclerosis, as well as to predict the risk of its clinical complications (coronary artery disease (CAD) or cardiovascular events) [1]. Increased cIMT and/or atheromatous plaque may increase the risk of cardiovascular disease by up to four-fold in comparison with individuals who do not suffer from carotid target organ damage (cTOD) [2–5]. To be more specific, with every increase of 0.1 mm of the cIMT, the risk of coronary heart disease is increased by 15% and the risk of cerebrovascular disease by 18% [2, 6–9].

The cIMT has been related to several components of the Mediterranean diet (MD) in isolation (fruits, whole grain cereals, fibre, walnuts and olive oil) [10, 11]. Nevertheless, its relationship with the adherence to the MD as a whole is uncertain. Thus, some studies suggest the MD may slow down the progress of the cIMT [12, 13], whereas others do not show this association, or just show it in individuals with a basal cIMT up to 0.9 mm [14, 15]. Diet quality indices address the diet’s complexity and are calculated by a combination of foods and/or nutrients which together represent a dietary pattern [16]. The best values in these indices have been associated with positive changes in weight [17]. They have also indicated an inverse relationship with several inflammatory response markers [18]. On the other hand, the diet quality has been related with vascular health assessed by arterial stiffness and endothelial dysfunction [19]. However, there is little evidence of the association between the diet quality indices and the surrogate markers of atherosclerosis as the cIMT. For all these reasons and taking into account that the cIMT shows a greater predictive value of cardiovascular disease [20], the current study analysed the relationship between the cIMT and the diet quality. It is assessed through the Diet Quality Index (DQI) questionnaire in adults.

## Methods

### Design

The findings shown here are a sub-analysis of the MARK study [21]. The MARK study is a cross-sectional study whose purpose was to evaluate if ankle-brachial index (ABI), measures of arterial stiffness by the Cardio ankle vascular index (CAVI), postprandial glucose, glycosylated haemoglobin, self-measured blood pressure and the presence of comorbidities are independently associated with the incidence of vascular events and whether they can improve the predictive capacity of current risk equations in the intermediate risk population. The second step will be 5- and 10-year follow up to evaluate cardiovascular morbidity and mortality.

### Study population

The MARK study included 2384 participants but only in 500 of these was carotid ultrasound performed. This was the only reason to exclude the rest (1884 participants) from the analysis of this work. The population comprised individuals aged between 35 and 74 years who had intermediate cardiovascular risk, which was defined as coronary risk between 5 and 15% at 10 years according to the Framingham-adapted risk equation (REGICOR) [22], cardiovascular mortality risk between 1 and 5% at 10 years according to the SCORE equation [23] or moderate risk according to the 2007 European Society of Hypertension guidelines for the management of arterial hypertension [24].

The exclusion criteria were terminal illness, institutionalization at the appointment time, or a personal history of atherosclerotic disease (Acute myocardial infarction, angina pectoris or stroke), registered in his/her electronic clinical history. Sample selection was done with a random sample population aged 35 to 74 (both included) who had an intermediate risk of a cardiovascular event. The population was recruited from three different regions of Spain (Catalonia, Baleares and Castilla y León) and data collection was carried out from July 2011 to June 2013.

The study was approved by the independent ethics committee of the Health Area of Salamanca (Spain) and all participants gave written informed consent according to the recommendations of the Declaration of Helsinki.

### Measurements

#### Assessment of the diet quality index (DQI)

Diet quality was evaluated by the diet quality index (DQI), which is derived from the short diet quality screener (SDQS). The SDQS is the only questionnaire validated in Spanish population that assesses diet quality [25]. The SDQS includes 18 food groups divided into three categories. Each category is scored with 1, 2 or 3 points, depending on the frequency of its consumption and whether the consumption of these products is considered beneficial to health (increased consumption, higher score) or detrimental to health (increased consumption, lower score). All food item scores are summed. The total possible score thus ranges from 18 to 54. A higher score suggests a higher diet quality. More details about the estimation of the DQI are presented in Table 1.

**Table 1:** Scoring method for the Diet Quality Index (DQI)

1. Daily frequency consumption of the following foods during the last 12 months				
Food	Amount	<1 time/day	1 time/day	≥2 times/day
Bread	1–2 slices	1	2	3
Vegetables/salad	1 serving	1	2	3
Fruit	1 piece of serving	1	2	3
Yoghurt or milk	1 tub/1 glass	1	2	3
Pasta or rice	1 serving	1	2	3
Oil (olive or sunflower)	1 tablespoon	1	2	3
Alcoholic beverages	1 drink	1	3	1
Breakfast flakes	1 bowl	1	2	3
2. Weekly frequency consumption of the following during the last 12 months				
Food	Amount	<4 times/week	4–6 times/week	≥7 times/week
Meat	1 serving	3	2	1
Sausages	1–3 slices	3	2	1
Cheese	1 serving	3	2	1
Pastry or sweets	1 piece or serving	3	2	1
Butter or lard	1 teaspoon	3	2	1
Other vegetable oils	1 tablespoon	3	2	1
Fast food	1 serving	3	2	1
3. Weekly frequency consumption of the following foods during the last 12 months				
Food	Amount	<2 times/week	2–3 times/week	≥4 times/week
Fish	1 serving	1	2	3
Legumes	1 serving	1	2	3
Nuts	1 handful	1	2	3

Source: Public Health Nutr 2012, 15(4):618–626

#### Common carotid artery intima media thickness (cIMT)

Carotid ultrasound to assess cIMT was performed by two investigators trained for this purpose before starting the study. A Sonosite Micromax ultrasound (Sonosite Inc., Bothell, Washington, USA) device paired with a 5–10 MHz multi-frequency high-resolution linear transducer with Sonocal software was used for automatic measurements of cIMT to optimize reproducibility. Measurements were made of the common carotid artery after examining a 10 mm longitudinal section 1 cm from the bifurcation. Measurements were performed at the proximal and distal wall in the lateral, anterior and posterior projections. They followed an axis perpendicular to the artery to discriminate two lines: −one for the intima-blood interface and the other for the media-adventitious interface. A total of six measurements were obtained for the right carotid and six measurements for the left carotid artery. We used the mean and the maximum cIMT values that were automatically calculated by the software [26]. The measurements were obtained with the subject lying down, with the head extended and slightly turned opposite of the carotid artery under study. The presence of a plaque was identified by a cIMT ≥1.5 mm or by a focal increase in thickness of 0.5 mm or 50% of the surrounding cIMT value. The existence of a plaque or a carotid cIMT >0.9 mm, was considered as cTOD [27].

#### Definition of hypertension, type 2 diabetes mellitus, dyslipidemia and obesity

The 2013 European Society of Hypertension and European Society of Cardiology guidelines defined hypertension as values ≥140 mmHg SBP and/or ≥90 mmHg DBP or the presence of antihypertensive treatment [28]. The guidelines of the American Diabetes Association defined diabetes mellitus type 2 as the presence of HbA1c ≥6.5% or fasting plasma glucose ≥126 mg/dl or 2 h plasma glucose ≥200 mg/dl during an oral glucose tolerance test or in a patient with classic symptoms of hyperglycemia or hyperglycemic crisis, a random plasma glucose ≥200 mg/dl or the presence of antidiabetic treatment [29]. The American Association of Clinical Endocrinologists’ guidelines for management of dyslipidemia and prevention of atherosclerosis, defined dyslipidemia as the presence of total cholesterol ≥240 mg/dl or triglycerides ≥200 mg/dl or the presence of lipid-lowering drugs [30]. Obesity was defined at values ≥30 kg/m^2^.

#### Other variables

Other variables collected including sociodemographic variables, pharmacological treatment, blood pressure measurement, laboratory tests and variables related to the lifestyles (smoking, alcohol consumption and physical activity) [21].

#### Statistical analysis

Descriptive statistics were expressed as mean ± standard deviation for continuous variables or number (%) for categorical variables. The Chi-square test or the Fisher’s exact test analysed the association between independent categorical variables. The difference in the means between 2-category variables was analysed using the independent samples Student’s t-test. We performed multiple linear regression analyses, including the cIMT (mean and maximum) as the dependent variables and the DQI index as independent variable in three models. We performed a first model unadjusted, a second model adjusted for age and gender and a third model adjusted also for level of education, smoking, physical activity, antihypertensive, antidiabetic and lipid lowering drugs. In the logistic regression analysis, we used the DQI as the independent variable, split into in two categories using the median value (40 points) as cut-off (DQI below than 40 points = 1; DQI ≥40 = 0). The dependent variables used were a cIMT > 0.9 mm, presence of atherosclerotic plaque or the presence of either one. The same adjustments as in the multiple linear regression analysis were used. In order to analyse the cIMT by tertiles of DQI, an ANCOVA test has been controlled by the covariates used in the regression models 2 and 3. For bilateral hypothesis contrasts, an alpha risk of 0.05 was set as the limit of statistical significance using SPSS v.23.0.

## Results

General, anthropometric and clinical characteristics of the participants are presented in Table 2. The age of the participants (mean ± SD) was 60.3 ± 8.4 years and 54.4% were male. Among the 500 participants, 80.2%, 27.6% and 83.6% were hypertensive, diabetics and dyslipidemics, respectively. The proportion of individuals treated with drugs was 53.2% for antihypertensives, 16.4% used antidiabetics and 37.0% with lipid lowering drugs. Systolic and diastolic blood pressure had higher values in men (*p* < 0.01) and heart rate was higher in women (*p* = 0.01). Mean values of vascular structure measurements were: cIMT (mean) 0.73 ± 0.09 mm, cIMT (maximum) 0.90 ± 0.11 mm, these are lower in women (*p* < 0.01). On the other hand, 83 (16.6%) subjects presented with plaque, and 85 (17.0%) had cTOD. The mean of the DQI score was 40.08 ± 2.79, with no differences observed between men and women.

**Table 2:** Clinical characteristics, lifestyles and carotid measurements of the study population

	OVERALL (*n* = 500)	MEN (*n* = 272; 54.4%)	WOMEN (*n* = 228; 45.6%)	p
Age (years)	60.3 ± 8.4	59.0 ± 8.5	61.9 ± 8.0	0.002
Level of education				<0.01
Higher education	114 (22.8)	78 (28.7)	36 (15.8)	
High school	153 (30.6)	95 (34.9)	58 (25.4)	
Primary studies	233 (46.6)	99 (36.4)	134 (58.8)	
Smoking (n, %)	107 (21.4)	64 (23.5)	43 (18.9)	0.229
Physical activity (METs/h/week)	3541 ± 3358	4175 ± 3977	2784 ± 2202	<0.01
BMI (Kg/m^2^)	28.3 ± 4.2	28.3 ± 3.5	28.2 ± 4.9	0.733
Systolic blood pressure (mmHg)	133.9 ± 16.6	136.3 ± 15.9	131. ± 17.0	0.004
Diastolic blood pressure (mmHg)	81.3 ± 10.8	82.9 ± 10.1	79.3 ± 11.2	0.003
Heart rate (bpm)	70.3 ± 11.2	69.1 ± 11.7	71.7 ± 10.5	0.010
Obesity, BMI ≥30 (n, %)	137 (27.4)	69 (25.4)	68 (29.8)	0.270
Hypertension (n, %)	401 (80.2)	226 (83.1)	175 (76.8)	0.002
Diabetes (n, %)	138 (27.6)	87 (32.0)	51 (22.4)	0.016
Dyslipidemia (n, %)	418 (83.6)	221 (81.39)	197 (86.4)	0.146
Antihypertensive (n, %)	266 (53.2)	146 (53.7)	120 (52.6)	0.857
Antidiabetics (n, %)	82 (16.4)	52 (19.1)	30 (13.2)	0.089
Lipid lowering drugs (n, %)	185 (37.0)	99 (36.4)	86 (37.7)	0.781
Diet				
Diet Quality Index (total score)	40.08 ± 2.79	40.28 ± 2.83	39.85 ± 2.74	0.087
Vascular structure measurements				
IMT (mean)	0.73 ± 0.09	0.75 ± 0.10	0.72 ± 0.08	0.002
IMT (maximum)	0.90 ± 0.11	0.92 ± 0.12	0.88 ± 0.10	0.003
Presence of plaque (n, %)	83 (16.6)	58 (21.3)	25 (11.0)	0.002
Carotid target organ damage (n, %)	85 (17.0)	59 (21.7)	26 (11.4)	0.003

*METs* metabolic equivalents, *BMI* body mass index, *bpm* beats per minute, *IMT* carotid intima media thickness

Categorical variables are expressed as n (%) and continuous variables as mean ± standard deviation

T-student test. p: statistically significant differences (*p* < 0.05)

In the multiple linear regression analysis, no significant association was found between DQI and cIMT (mean) in the Model 3 adjusted by age, sex and other covariables (*p* = 0.690) (Table 3).

**Table 3:** Multiple linear regression analysis: Relationship between diet quality index and carotid intima media thickness

	IMT mean			IMT maximum		
	β unstandardized	95% CI	p	β unstandardized	95% CI	p
DQI (Total score)						
Model 1	0.001	−0.002 to 0.004	0.435	0.001	−0.002 to 0.005	0.453
Model 2	−0.001	−0.004 to 0.001	0.327	−0.002	−0.005 to 0.002	0.358
Model 3	−0.001	−0.003 to 0.002	0.690	−0.001	−0.004 to 0.003	0.703

*DQI* diet quality index, *IMT* carotid intima media thickness

β: regression coefficient; CI: confidence interval

p: statistically significant differences (*p* < 0.05)

Model 1: No adjustment

Model 2: Adjusted for age and sex

Model 3: Adjusted for age, sex, level of education, smoking, physical activity, antihypertensive, antidiabetics and lipid lowering drugs

Independent variable: Diet quality index

The logistic regression analysis (Table 4) did not show any relation between DQI and carotid atherosclerosis. The Model 3, adjusted by age, sex and other confounders, showed no association of DQI with cIMT > 0.9 mm (*p* = 0.890), nor with the presence of plaques (*p* = 0.799) or carotid TOD (*p* = 0.942).

**Table 4:** Logistic regression analysis: Relationship between diet quality index and carotid target organ damage

	IMT >0.9 mm			Plaque			Carotid TOD		
	OR	95% CI	p	OR	95% CI	p	OR	95% CI	p
DQI									
Model 1	1.005	0.416 to 2.430	0.991	0.914	0.566 to 1.474	0.711	0.925	0.576 to 1.487	0.749
Model 2	1.115	0.451 to 2.757	0.814	0.994	0.609 to 1.624	0.982	1.019	0.626 to 1.658	0.940
Model 3	0.934	0.351 to 2.485	0.890	0.935	0.556 to 1.572	0.799	0.981	0.584 to 1.648	0.942

*IMT* intima media thickness, *TOD* target organ damage, *DQI* diet quality index, *OR* odds ratio, *CI* confidence interval

It was considered as carotid TOD if exists a plaque or a carotid IMT >0.9 mm. Presence of a plaque was identified by an IMT ≥1.5 mm or by a focal increase in thickness of 0.5 mm or 50% of the surrounding carotid IMT value

p: statistically significant differences (*p* < 0.05)

Model 1: No adjustment

Model 2: Adjusted for age and sex

Model 3: Adjusted for age, sex, level of education, smoking, physical activity, antihypertensive, antidiabetics and lipid lowering drugs

Dependent variables IMT (IMT < 0.9 mm = 0; IMT > 0.9 mm = 1); Plaque (No = 0, Yes = 1), Carotid TOD (No = 0, Yes = 1)

Independent variable: DQI two categories (DQI < 40 = 1; DQI ≥ 40 = 0)

**Fig. 1:**
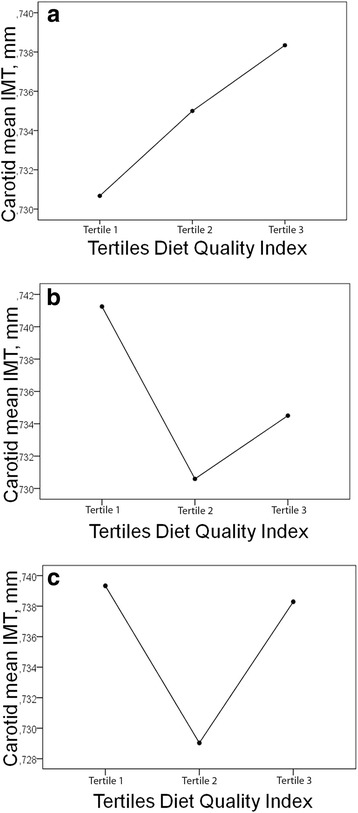
cIMT (mean) according to the DQI tertiles by ANCOVA test. DQI tertiles (T1 < 39; T2 39 to 41; T3 > 41). **a** Model 1 unadjusted (*p* = 0.783). **b** Model 2 adjusted for age and sex (*p* = 0.520). **c** Model 3 adjusted for age, sex, level of education, smoking, physical activity, antihypertensive, antidiabetics and lipid lowering drugs (*p* = 0.458)

Figure 1 shows the values of the carotid mean cIMT by DQI tertiles (T1 < 39; T2 39 to 41; T3 > 41). After adjusting by age and sex and other confounders (Model 3), no association was found between DQI score and cIMT, being the differences between the DQI tertiles no significant (*p* = 0.458).

## Discussion

The results of this study show no association of the DQI with the cIMT, the presence of atherosclerotic plaques or the cTOD. These findings suggest that food quality indices may not be so useful as predictors of surrogate atherosclerosis markers as carotid damage. These results support the evidence available to date.

There is growing evidence of the relationship between cIMT with both dietary habits and individual dietary components. However, the results are inconclusive depending on whether one considers the nutritional components in isolation or consumed within a dietary pattern. The cIMT has been linked with food groups. Thus, an association between greater intake of fruit with lower cIMT has been found, especially in high cardiovascular risk individuals [11, 31, 32]. However, testing a possible association with the intake of vegetables and whole grains showed contradictory results [12, 33, 34]. Among nutrients, an inverse relationship between the ratio of poly-unsaturated to saturated fatty acid with the cIMT has been reported [35, 36]. However, the evidence is still inconsistent in relation to sodium intake, n6, n3 and other micronutrients [37]. In a similar manner, the relationship between cIMT with dietary components consumed together is unclear. Some studies have demonstrated an inverse association between dietary patterns and cIMT [12, 38, 14]. Mikkilä et al. [39], as part of the Cardiovascular Risk in Young Finns Study, determined that traditional dietary pattern (characterized by high consumption of rye, potatoes, butter, sausages, milk and coffee) was independently associated with cIMT in men. However, the health-conscious dietary pattern scores (characterized by a high consumption of vegetables, legumes and nuts, rye, tea, cheese and other dairy products) showed no significant associations with cIMT. Moreover, consumption of the Mediterranean diet, closely associated with cardiovascular health [40], has been inversely associated with cIMT though only in a population with high cardiovascular risk (cIMT >0.9 mm) [14].

The association between diet quality indices and subclinical atherosclerosis has been hardly studied. Hoebeeck et al. [41] have also studied this relationship in 2524 middle-aged adults with low cardiovascular risk of the Asklepios study and they have only demonstrated a relationship between dietary diversity score and the presence of femoral atherosclerosis, but not with the dietary quality score. Nevertheless these authors have not found any significant association, neither with cIMT nor with the presence of plaques. These findings are consistent with those obtained in our study, with a broad sample of participants with intermediate cardiovascular risk.

Some of the possible explanations why high DQI score diet failed to improve cIMT level in intermediate cardiovascular risk subjects should be addressed. It is necessary to take into account that generally nutrients are consumed in combination with others and not as isolated elements. Although the evidence points to a beneficial relation of certain nutritional components on cIMT, and therefore on subclinical atherosclerosis, this relationship is unclear when the same elements are taken with others, either in a specific food group or in the context of dietary patterns considered to be healthy. This lack of clear evidence in the relationship between c-IMT with both elements considered healthy or unhealthy could counteract the possible beneficial effect of the first by the possible detrimental effect of the latter. The short diet quality screener (SDQS), gives the highest score (3 points) to a high consumption of foods that have proven beneficial effect on cIMT [11, 31, 32] but uses the same scoring criteria for consumption of other products whose evidence is not sufficiently contrasted [12, 33, 34]. On the other hand, this relationship (cIMT-dietary habits) has been shown in individuals at high cardiovascular risk (cIMT >0.9 mm) [14]. However, it remains unclear in subjects with medium or low cardiovascular risk. The European Guidelines on Cardiovascular disease prevention in clinical practice highlights the importance in those (intermediate cardiovascular risk) subjects of promoting healthy lifestyle behaviour by tackling unhealthy lifestyles (e.g. poor-quality diet, physical inactivity, smoking) and by optimising risk factors [42, 43]. More prospective studies are therefore needed to assess this relationship in selected and more diverse populations.

The main limitation of this study is its cross-sectional design, which prevents any causal relationship between diet quality and carotid atherosclerosis being elucidated. More longitudinal studies and clinical trials are needed to explore this association. The assessment of quality index was mainly based on the DQI questionnaire, which was designed to assess the habitual diet by asking about the frequency of a limited number of food items. In spite of the fact that DQI shows a reasonable construct validity, it is important to note that it is based on the report of the habitual intake of 18 specific food items, hence some food items that may have an influence (either positive or negative) on cIMT could be not included in this questionnaire. It has also to be taken into account the role of the memory and the interpretation of the questions by the respondents.

## Conclusions

The diet quality index is not associated with the carotid subclinical atherosclerosis in intermediate cardiovascular risk individuals.

## Abbreviations

cIMT: Common carotid artery intima media thickness
cTOD: Carotid target organ damage
DQI: Diet quality index
MD: Mediterranean diet
